# An aging-susceptible circadian rhythm controls cutaneous antiviral immunity

**DOI:** 10.1172/jci.insight.171548

**Published:** 2023-10-23

**Authors:** Stephen J. Kirchner, Vivian Lei, Paul T. Kim, Meera Patel, Jessica L. Shannon, David Corcoran, Dalton Hughes, Diana K. Waters, Kafui Dzirasa, Detlev Erdmann, Jörn Coers, Amanda S. MacLeod, Jennifer Y. Zhang

**Affiliations:** 1 Department of Dermatology,; 2 Department of Molecular Genetics and Microbiology,; 3 Department of Immunology,; 4 Duke Center for Genomic and Computational Biology,; 5 Department of Neurobiology,; 6 Department of Psychiatry and Behavioral Sciences,; 7 Department of Biomedical Engineering, and; 8 Department of Neurosurgery, Duke University, Durham, North Carolina, USA.; 9 Howard Hughes Medical Institute, Chevy Chase, Maryland, USA.; 10 Department of Surgery, Division of Plastic, Maxillofacial, and Oral Surgery, and; 11 Department of Pathology, Duke University, Durham, North Carolina, USA.

**Keywords:** Aging, Dermatology, Cellular immune response, Innate immunity, Skin

## Abstract

Aged skin is prone to viral infections, but the mechanisms responsible for this immunosenescent immune risk are unclear. We observed that aged murine and human skin expressed reduced levels of antiviral proteins (AVPs) and circadian regulators, including Bmal1 and Clock. Bmal1 and Clock were found to control rhythmic AVP expression in skin, and such circadian control of AVPs was diminished by disruption of immune cell IL-27 signaling and deletion of Bmal1/Clock genes in mouse skin, as well as siRNA-mediated knockdown of CLOCK in human primary keratinocytes. We found that treatment with the circadian-enhancing agents nobiletin and SR8278 reduced infection of herpes simplex virus 1 in epidermal explants and human keratinocytes in a BMAL1/CLOCK-dependent manner. Circadian-enhancing treatment also reversed susceptibility of aging murine skin and human primary keratinocytes to viral infection. These findings reveal an evolutionarily conserved and age-sensitive circadian regulation of cutaneous antiviral immunity, underscoring circadian restoration as an antiviral strategy in aging populations.

## Graphical abstract

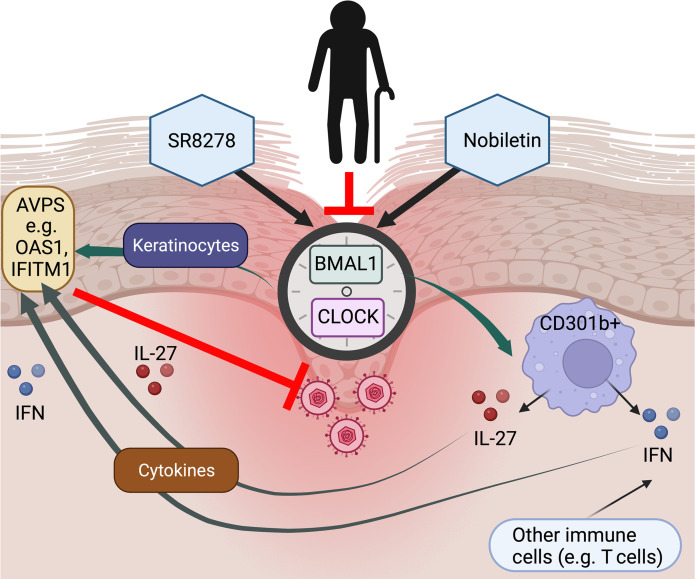

## Introduction

The skin acts as a physical barrier to invading pathogens, which can be disrupted by genetic defects, environmental challenges, wounds, and microinjuries (1). Skin barrier disruptions are of special concern for elderly patients because of the reduced regenerative capacity in aged skin. Consequently, these patients experience an increased risk of pathogen infection and other clinical issues (2, 3). Nevertheless, how an aged skin microenvironment affects barrier function and immunosenescence is not well understood. Aspects of the skin microenvironment that influence barrier defense include location of disruption, microbial content, moisture status, and age of the skin (3). In addition, the time at which a wound is inflicted changes barrier responses and results in differential healing rates (4), suggesting that the circadian rhythm in skin regulates tissue regeneration and immune responses.

The circadian rhythm controls time-of-day biological responses and regulates components of cell proliferation and wound reepithelialization (5). Mice deficient in Bmal1, a core transcription factor of the circadian clock, exhibit greater burden in viral infections (6, 7), indicating that circadian rhythms influence antiviral functions. Circadian function declines in older individuals (8); however, to our knowledge, aging circadian rhythms have not previously been characterized in the context of immunosenescent cutaneous barrier defenses.

IL-27, a member of the IL-12 family of heterodimeric cytokines, was recently implicated in cutaneous defense against Zika virus (9). In response to skin injury, CD301b^+^ leukocytes are rapidly recruited to the wound site (10) and produce IL-27, which subsequently potentiates wound closure and induces production of innate antiviral proteins (AVPs) (9, 11). AVPs encompass several families, including oligoadenylate synthetase (OAS1, OAS2, OAS3), myxovirus-resistance proteins (MX1 and MX2), and IFN-induced transmembrane (*IFITM*) family proteins (1). Circadian rhythms have been implicated in IFN-stimulated gene responses in several tissues, including skin and lung (12, 13). However, it is unclear if skin barrier antiviral function is influenced by circadian rhythms.

In this study, we discovered that levels of circadian factors Bmal1 and Clock decrease in aged skin. We also found that circadian dysregulation impairs cutaneous AVP expression via epidermal keratinocyte-autonomous and leukocyte-derived cytokine-mediated processes. Our studies show that murine cutaneous AVPs are regulated by Bmal1 and Clock in intact and wound states. We found that circadian mutant and aged mice exhibited reduced IL-27 expression in skin wounds. Additionally, IL-27, along with type I IFN signaling, is required for time-of-day–dependent circadian regulation of wound-induced AVP production. Here, we demonstrate that genetic loss of function of circadian factors sensitized skin and keratinocytes to herpes simplex virus 1 (HSV-1) infection, whereas agents such as SR8278 and nobiletin that increase circadian rhythm amplitudes in other tissues (14) enhanced cutaneous circadian rhythms and reduced HSV-1 viral burden in human keratinocytes and epidermal explants. Finally, we demonstrate that these circadian agents have antiviral effects in aging murine skin and human skin cells.

## Results

### Aging skin displays a decline in circadian rhythm and antiviral immune function.

**Figure 1:**
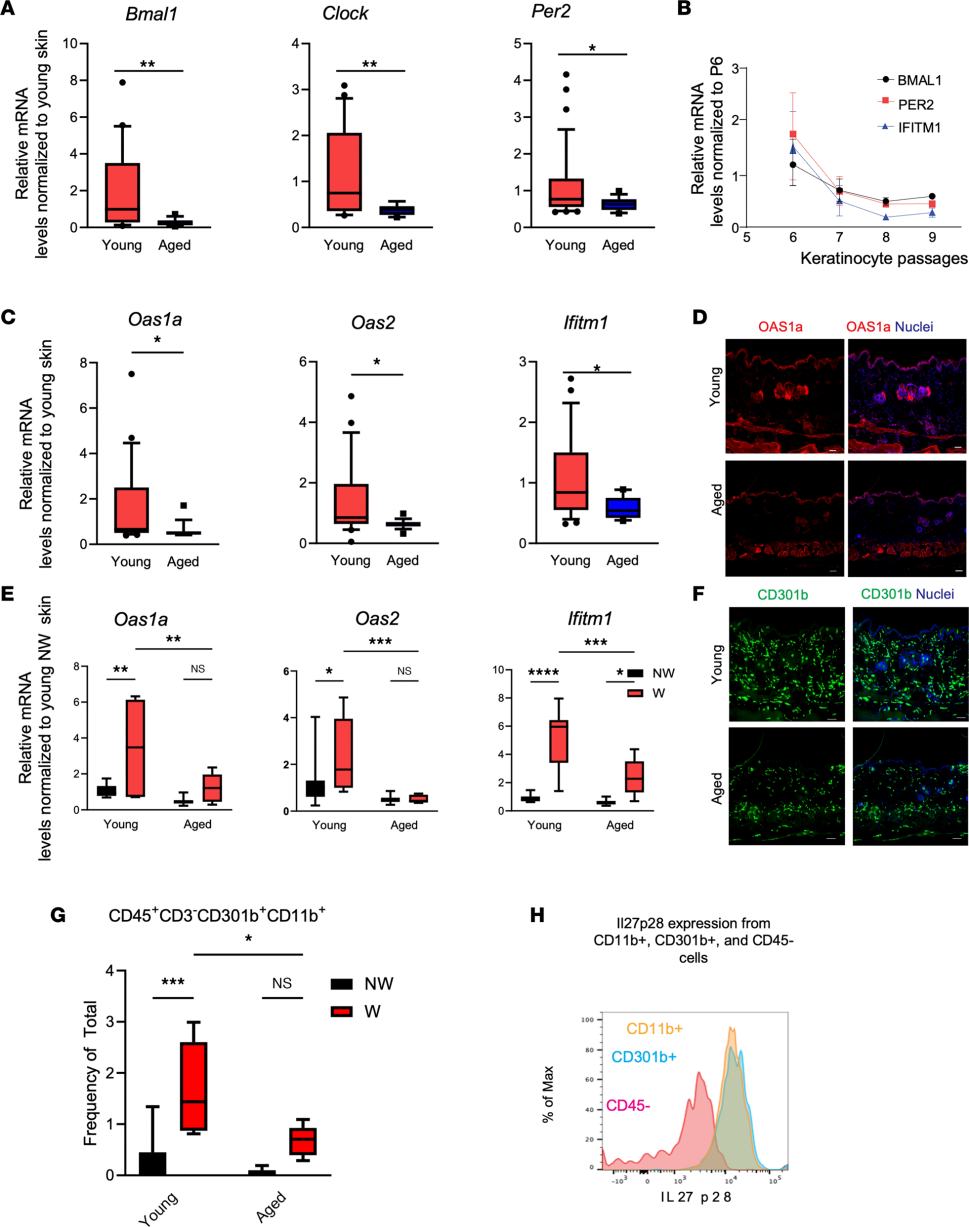
Aging skin exhibits diminished circadian, AVP, and IL-27 transcription. (**A**) Quantitative PCR (qPCR) of *Bmal1*, *Clock*, and *Per2* in aged (*n* = 14–16, >1 year old) and young (*n* = 32–34 1 month old) male murine skin. Graphs represent averages of relative mRNA ± SEM with GAPDH used for an internal control. *P* values were obtained via 2-tailed Student’s *t* test. (**B**) qPCR of *BMAL1*, *PER2*, and *IFITM1* in human primary keratinocytes over serial passaging (*n* = 2–3 donors/passage). Graphs represent averages of relative mRNA ± SEM with GAPDH used for an internal control. (**C**) qRT-PCR of *Oas1*, *Oas2*, and *Ifitm1* in aged and young murine back skins as described in **A**. *P* values were obtained via 2-tailed Student’s *t* test. (**D**) Immunostaining for OAS1a (orange) and nuclei (blue) in aged and young nonwounded skin. Scale bar: 25 μm. (**E**) qRT-PCR of AVP in young and old skin 24 hours after wounding. Graphs represent averages of relative mRNA ± SEM with GAPDH used for an internal control. *P* values were obtained via 2-way ANOVA with multiple comparison. NW, nonwounded skin; W, wounded skin. (**F**) Immunostaining for CD301b (green) and nuclei (blue) in aged and young unwounded skin. Scale bar: 25 μm. (**G**) Flow cytometry showing reduced numbers of CD301b^+^ cells in aged skin compared with young skin (*n* = 4 mice/group) as a percentage of total harvested live, single cells. *P* values were obtained via 2-way ANOVA with multiple comparisons. (**H**) Histogram displays IL-27 production from CD11b^+^ (yellow) and CD301b^+^ (blue) cells compared with CD45^–^ (red). The flow cytometry gating strategy is included in Supplemental Figure 1. *P ≤ 0.05, **P ≤ 0.01, ***P ≤ 0.001, ****P ≤ 0.0001.

The link between aging, cutaneous circadian rhythms, and barrier defense is unclear. To address this, we first investigated expression of circadian factors in murine skin of varying ages. We found that *Bmal1*, *Clock*, and *Per2* were downregulated in aged (older than 12 months) skin compared with young (approximately 3–6 months) skin (Figure 1A). Serial passaging of primary human keratinocytes, which acts as a surrogate for human skin aging (15), showed that circadian transcriptional activity decreased with increasing passage numbers (Figure 1B). This is also corroborated in an existing human skin data set, where ARNTL (BMAL1) appears to peak in middle age before declining in expression (16).

We next investigated whether aging skin exhibits deficiency in AVP production. Using quantitative real-time PCR (qRT-PCR) and immunofluorescence, we found that mRNA and protein levels of AVPs (*Oas1a*, *Oas2*, *Ifitm1*) were significantly reduced in aged murine skin compared with young skin (Figure 1, C and D) and visualized in epidermal structures and sebaceous glands (17). Barrier disruption triggers an AVP response in young skin (9, 11, 18). We observed that wounding significantly elevated AVP induction at 24 hours after wounding in young and old skin; however, the magnitude of induction was significantly higher in wounds of young skin compared with that of aged skin (Figure 1E).

AVPs are induced in the skin wound microenvironment by IL-27, a cytokine produced by CD301b^+^ leukocytes of the skin (9, 11). To address if aging affects CD301b^+^ signaling, we examined murine skin across ages for the presence of CD301b^+^ cells via immunostaining and found that CD301b^+^ cells were reduced in the skin of aged compared with younger mice (Figure 1F). Flow cytometry analysis revealed that wounded aged skin had a decreased influx of CD301b^+^ cells and expression of IL-27 (Figure 1, G and H) (the gating strategy is shown in Supplemental Figure 1; supplemental material available online with this article; https://doi.org/10.1172/jci.insight.171548DS1). IL-27 works in concert with type I IFN in inhibiting Zika virus infection (9). To test if type I IFN signaling is also affected by aging, we performed qRT-PCR comparing the expression levels of type I IFNs and their receptor *Ifnar1* in wounds of young and aged mice. We found no differences in *Ifna2*, *Ifnβ*, *Ifna4*, and *Ifna11* between old and young skin-wound samples collected 24 hours after wounding (Supplemental Figure 2). These data supported a link among aging, cutaneous rhythms, and antiviral barrier defense.

### Cutaneous circadian rhythms regulate AVPs.

**Figure 2:**
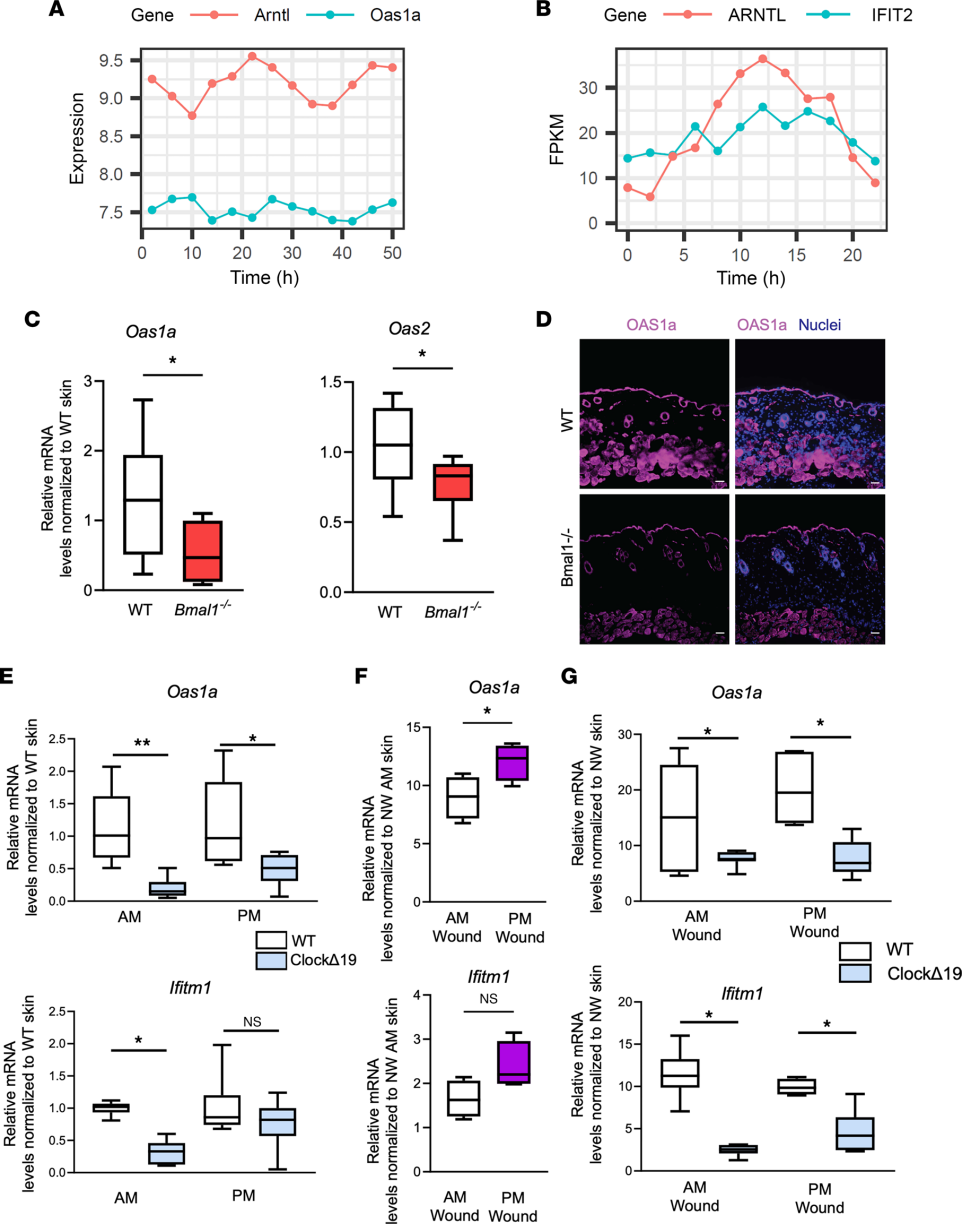
Circadian rhythm transcriptional networks include antiviral genes in mammalian skin. (**A** and **B**) Line plots showing rhythmic expression of circadian factors and AVP genes in (**A**) murine skins (GSE38625) and (**B**) baboon skins (GSE98965). Heatmaps including additional genes can be found in Supplemental Figure 3. (**C**) qRT-PCR of *Oas1a* and *Oas2* in intact skin of *Bmal1^–/–^* mice and WT littermates. (*n* = 3 mice/group, with technical triplicates per mouse). Graphs represent averages of relative mRNA ± SEM with GAPDH used for internal control. (**D**) Immunostaining for OAS1a (purple) in WT and *Bmal1^–/–^* skin. Nuclei are stained blue. Scale bar: 25 μm. (**E**) qRT-PCR of *Oas1a* and *Ifitm1* in intact belly skin of *ClockΔ19* mice and BALB/C WT littermates harvested at 5 am (AM wound) or 5 pm (PM wound). (**F**) qRT-PCR of *Oas1* and *Ifitm1* in skin wounds of C57BL/6 inflicted at times indicated and harvested 24 hours later (*n* = 4 mice/group). For *Ifitm1*, *P* = 0.0725. (**G**) qRT-PCR of *Oas1a* and *Ifitm1* in skin wounds of *ClockΔ19* mice and BALB/C WT littermates inflicted at the indicated time and harvested 24 hours later. (**E** and **G**) *n* = 3 mice/group with technical triplicates, except WT pm time group, which used 2 mice. *P* values reported in this figure were obtained via 2-tailed Student’s *t* test. *P ≤ 0.05, **P ≤ 0.01. FPKM, fragments per kilobase of transcript per million mapped reads.

Next, we asked if circadian decline could mechanistically be responsible for this immunosenescent barrier defense. To test the circadian-innate immunity link in the context of skin, we queried published microarray gene expression data of murine skin harvested every 4 hours (19). We scaled and clustered the expression of AVPs across Zeitgeber (standardized time of day) time points within a 24-hour span using an additional data set (19) and separated the genes into 5 clusters of distinct expression profiles. We found a variety of antiviral genes whose pattern of expression coincided with *Bmal1* (*Arntl*) in murine skin (Supplemental Figure 3). One such gene, *Oas1a*, had a temporal expression pattern coincident with *Arntl* changes (Figure 2A). We then used a circos plot and determined a broader time-of-day regulation of antiviral immune genes in the skin (Supplemental Figure 3). This was further supported by findings in baboon skin (20) (Supplemental Figure 3), where a number of antiviral genes, including *IFIT2*, had rhythmic expression similar to *ARNTL* (Figure 2B).

To validate these computational data and test if these AVP fluctuations are linked to circadian factors, we harvested belly skin from WT and *Bmal1^–/–^* C57BL/6J mice at 8 am and 8 pm. We found that the basal expression of AVPs varied in the skin, as shown by qRT-PCR (Supplemental Figure 3). In agreement with these data, AVPs were reduced in *Bmal1^–/–^* skin compared with WT skin as measured via qRT-PCR for *Oas1a* and *Oas2* (Figure 2C) and immunostaining for Oas1a (Figure 2D). Similar results were obtained by qRT-PCR in the skin of circadian deficient ClockΔ19-mutant mice (21), where circadian mutant mice expressed less AVP than did their WT littermates (Figure 2E).

To further examine the link between circadian genes and AVPs in the context of barrier disruption, we first compared AVP induction between WT skin-wound samples collected at 8 am and 8 pm, 24 hours after wounding. *Oas1a* expression was significantly higher in 8 pm wounds than that of 8 am wounds (Figure 2F); *Ifitm1* exhibited a similar trend, though it did not reach significance. Compared with WT counterparts, ClockΔ19 mutant skin had a significant decrease in wound-induced AVP production between 5 a.m. and 5 p.m. time points (Figure 2G). These data supported a link between circadian rhythms and AVPs of the skin.

### Circadian regulation of AVP induction requires IL-27 and type I IFN signaling.

**Figure 3:**
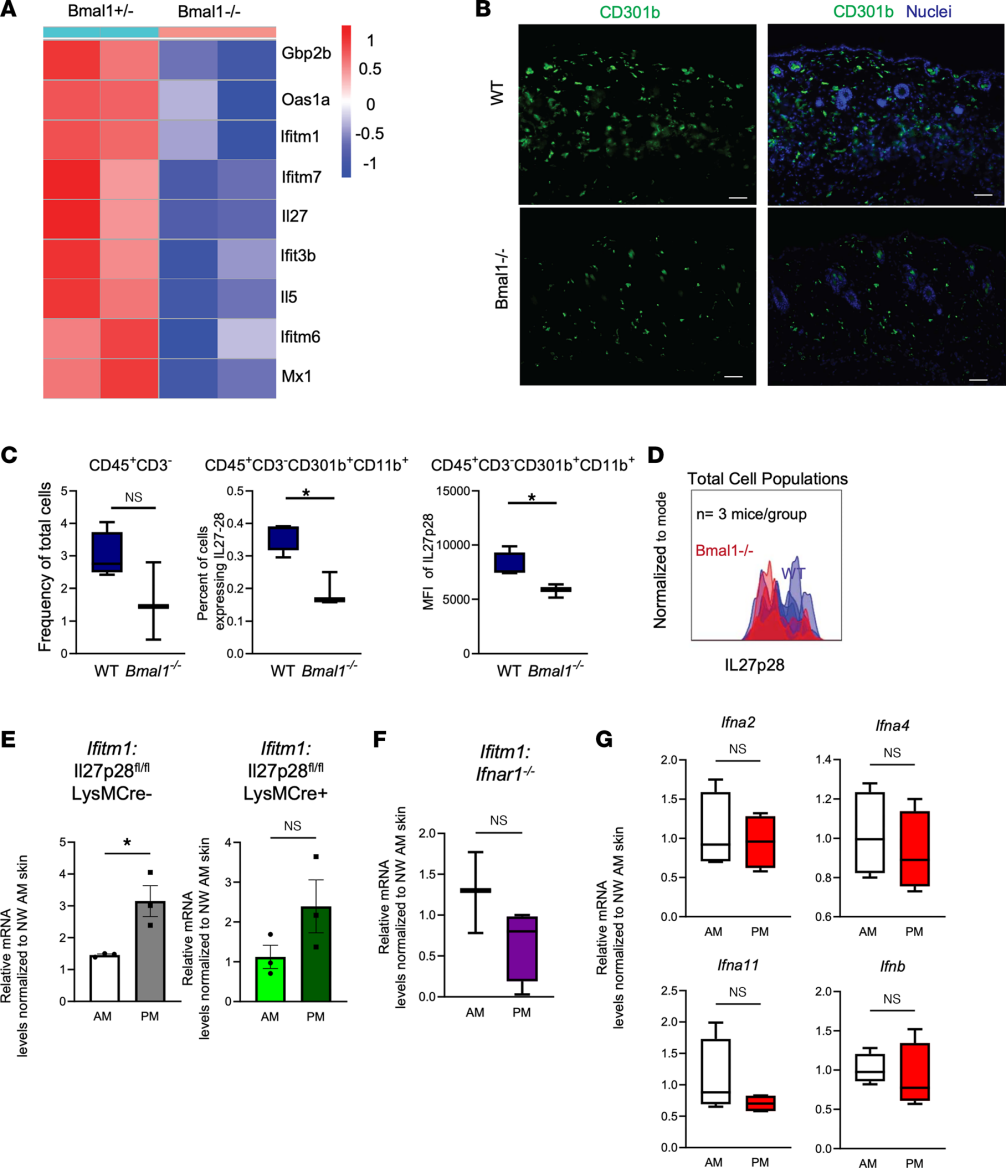
Circadian rhythms of wound-induced AVPs require cytokine signaling. (**A**) Heatmap of microarray expression of *Bmal1^–/–^* compared with heterozygotes intact murine skin as measured at Zeitgeber time 22 (GSE38625). (**B**) Immunostaining for CD301b (green) and nuclei (blue) in WT and *Bmal1^–/–^* intact skin. Scale bar: 25 μm. Skin samples are the same sections displayed in Figure 2C. (**C**) Flow cytometry of CD301b^+^ cells expressing IL-27 as a percentage of total harvested live single cells and MFI of Il27p28 in *Bmal1^–/–^* skin and WT skin (*n* = 3–4 mice/group). (**D**) Histogram displays IL27p28 expression in *Bmal1^–/–^* (red line) and WT (blue line) mouse skin. The gating strategy is shown in Supplemental Figure 4. (**E**) qRT-PCR of *Ifitm1* in skin wounds of WT or *LysM-*Cre.*IL27p28^fl/fl^* mice (*n* = 3 mice/group). (**F**) qRT-PCR of *Ifitm1* in skin wounds of *Ifnar1^–/–^* mice (*n* = 3–4 mice). (**G**) qRT-PCR of type I IFNs in WT C57BL6 mice wounded at 8 am or 8 pm (*n* = 4 mice/time point), as in (**E**) above. Graphs represent averages of relative mRNA ± SEM with GAPDH used for an internal control. *P* values reported in this figure were obtained via 2-tailed Student’s *t* test. *P ≤ 0.05. NW, nonwounded skin.

Cytokine production and leukocyte trafficking are well-characterized immune phenotypes with a circadian level control (22). We examined IL-27 expression in *Bmal1^–/–^* and *Bmal1*^+/–^ mouse skin, aged around 1 month, in an existing data set (19). We noted decreased expression of IL-27 along with other IFN-responsive antiviral genes such as *Oas1a*, *Ifitm1*, *Ifitm7*, and *Ifit3b* in intact *Bmal1^–/–^* skin (Figure 3A). Immunostaining revealed that numbers of CD301b^+^ cells were reduced for intact *Bmal1^–/–^* skin compared with WT counterparts (Figure 3B). Flow cytometry analysis verified the decrease of CD301b^+^ cells in *Bmal1^–/–^* skin wounds and a reduced median fluorescence intensity (MFI) of IL-27p28 in these cells (Figure 3, C and D, and Supplemental Figure 4). To determine whether IL-27 is required for circadian regulation of AVP induction, we used a Cre-*loxP* mouse model to ablate *Il27p28* in lysozyme M–expressing (LysM-Cre) myeloid cells, including CD301b^+^ cells (11). We observed that deletion of IL-27 in myeloid cells markedly diminished the time-of-day response of wound induction of AVPs (Figure 3E and Supplemental Figure 5).

To test if type I IFN has a circadian wound effect, we wounded *Ifnar1^–/–^* mice at 8 am and 8 pm and found that *Ifnar1* loss blunted the significance of temporal variation in AVP expression (Figure 3F and Supplemental Figure 5). However, transcription of type I IFNs, including *Ifna2*, *Ifna4*, *Ifna11*, and *Ifnb*, did not change with respect to time of day when measured 24 hours after wounding (Figure 3G). These data supported a leukocyte mechanism of action for circadian AVP function.

### Keratinocyte-autonomous circadian rhythm regulates AVP transcription and cutaneous defense against HSV-1 infection.

**Figure 4:**
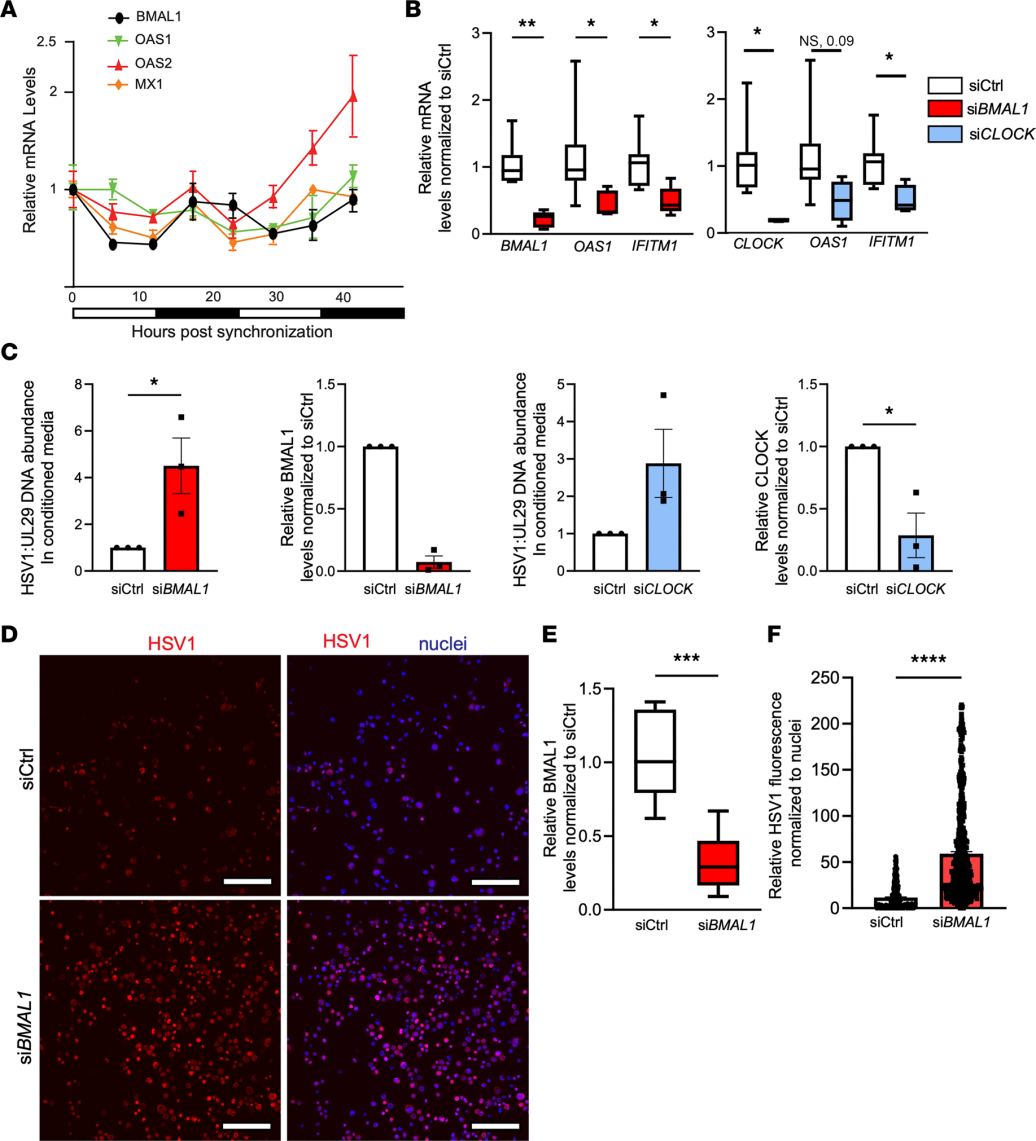
Keratinocyte autonomous circadian rhythm regulates antiviral activity. (**A**) qRT-PCR of *BMAL1*, *OAS1*, *OAS2*, and *MX1* in human primary keratinocytes synchronized via growth factor starvation and harvested every 6 hours (representative of 3 independent experiments). Graphs represent relative mRNA ± SEM with GAPDH used for an internal control and relative to that of 0-hour time point. (**B**) qRT-PCR of *BMAL1*, *CLOCK*, *OAS1*, and *IFITM1* in *NTERT* keratinocytes transfected with siRNA: si*Ctrl*, si*CLOCK*, or si*BMAL1* (*n* = 3 biological replicates). Graphs represent averages of relative mRNA ± SEM with GAPDH used for an internal control. (**C**) qPCR of HSV-1 gene UL29 in cell culture–conditioned medium of primary keratinocytes transfected with si*Ctrl*, si*BMAL1*, or si*CLOCK* 24 hours prior to infection with HSV-1 (MOI, 0.01). Knockdown efficacy is shown by qRT-PCR. Graphs represent averages of either relative DNA or mRNA ± SEM with GAPDH used for an internal control. Primary keratinocytes from donors were pooled (*n* = 3). (**D**) Immunofluorescence of HSV-1 (MOI, 0.01) in human *NTERT* keratinocytes transfected with si*Ctrl* or si*BMAL1*. Scale bar: 160 μm. (**E** and **F**) Knockdown efficacy of *BMAL1* in human *NTERT* cells is shown by qRT-PCR. ImageJ (Fiji) quantification of relative viral immunofluorescence normalized to nuclear staining. Box-and-whisker plots represent averages of relative immunofluorescence ± SEM (*n* = 3 samples/group in **E**; *n* > 400 cells/condition in **F**). *P* values reported in this figure were obtained via 2-tailed Student’s *t* test. *P ≤ 0.05, **P ≤ 0.01, ***P ≤ 0.001, ****P ≤ 0.0001.

Cell-autonomous immune defects are present in circadian deficient fibroblast cultures with respect to viral infection (6, 7), but it is unclear if other skin cells contribute to this phenotype. We asked whether keratinocyte-autonomous circadian rhythms contribute to the observed AVP regulation. To address this question, we synchronized circadian clocks of primary human epidermal keratinocyte cultures via an overnight incubation with omission of growth factor supplements. As expected, clock synchronization induced an oscillatory pattern of *BMAL1* expression (Figure 4A), coinciding with the oscillation of *OAS1*, *OAS2*, and *MX1* antiviral genes that approximate a cosinor sine model (Supplemental Figure 6). To establish a direct link between circadian factors and AVP expression, we performed siRNA-mediated knockdown of *BMAL1* (si*BMAL1*) and *CLOCK* (si*CLOCK*) in an immortalized *NTERT* keratinocyte culture. By qRT-PCR, we found that gene silencing of *BMAL1* and *CLOCK* significantly reduced expression of AVPs (Figure 4B). Furthermore, this effect had direct effects on viral replication. When primary keratinocytes with si*BMAL1* and si*CLOCK* were infected with HSV-1, they produced more virus than nonsilenced control (siCtrl) keratinocytes as measured by PCR of viral gene UL29 (Figure 4C). This was corroborated via immunofluorescence using *NTERT* keratinocytes, which showed circadian disruption was associated with significantly increased HSV-1 antigen levels (Figure 4, D–F).

### Circadian enhancement leads to decreased HSV-1 infection in the skin.

**Figure 5:**
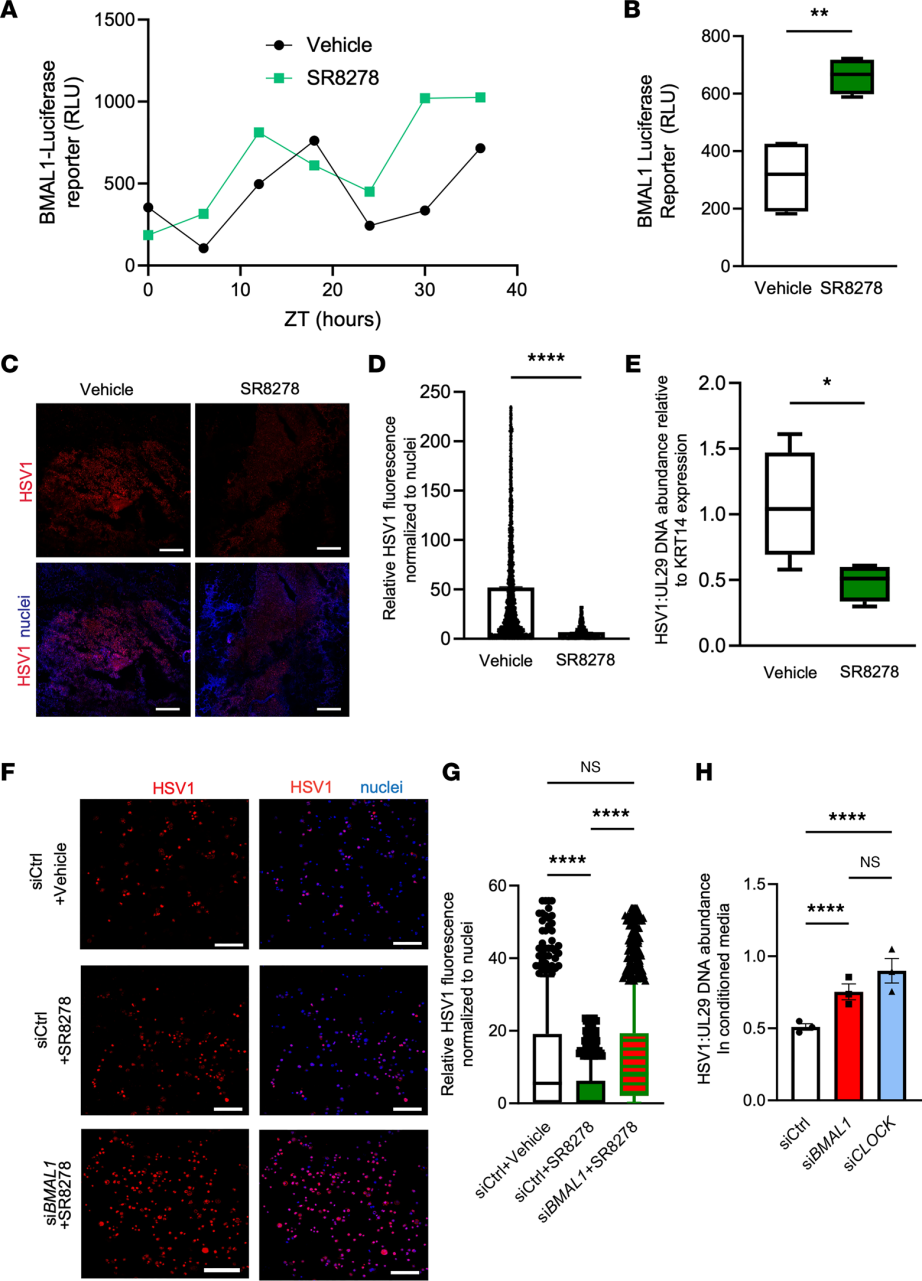
Pharmacological augmentation of circadian rhythm reduces HSV-1 infection in a BMAL1/CLOCK-dependent manner. (**A** and **B**) *Bmal1*–luciferase reporter assay. Human *NTERT* keratinocytes were transduced with *Bmal1*–luciferase reporter construct and starved of growth supplement overnight before treatment with vehicle or 10 μM SR8278. Cells were harvested (**A**) every 6 hours and (**B**) at 24 hours for RLU measurements (*n* = 4 samples). (**C**) Immunostaining for HSV-1 antigen in human epidermal explants infected with HSV-1 and treated with vehicle or 10 μM SR8278. Scale bar: 500 μm. (**D**) ImageJ (Fiji) quantification of viral immunofluorescence normalized to nuclear staining (*n* > 5,000 cells quantified per condition). (**E**) qPCR of HSV-1 viral gene UL29 relative to human KRT14 in epidermal skin infection (*n* = 4 skin explants). (**F**) Immunostaining for HSV-1 antigen in human *NTERT* keratinocytes (MOI, 0.01) transfected with si*Ctrl* or si*BMAL1* and supplemented with vehicle or 10 μM SR8278. Scale bar: 160 μm. (**A**–**F**) *P* values were obtained via 2-tailed Student’s *t* test. (**G**) ImageJ (Fiji) quantification of relative viral immunofluorescence normalized to nuclear staining. Data for quantification of siCtrl (vehicle) are the same as used in Figure 4E (*n* > 400 cells/condition quantified). (**G**–**H**) *P* values were obtained using 1-way ANOVA with multiple comparisons. (**H**) qPCR of HSV-1 viral gene UL29 in cell culture–conditioned medium of primary keratinocytes (MOI, 0.01) transfected with si*BMAL1* or si*CLOCK* and infected with HSV-1 (*n* = 3). Graphs represent averages of relative viral DNA or viral protein immunofluorescence normalized to human KRT14 or nuclear staining ± SEM. *P* values were obtained using 1-way ANOVA with multiple comparisons. *P ≤ 0.05, **P ≤ 0.01, ****P ≤ 0.0001. ZT, Zeitgeber time.

We hypothesized that enhancement of circadian function increases antiviral immunity of the skin. We expressed a *Bmal1* promoter–driven luciferase reporter (23) in *NTERT* keratinocytes and validated that *Bmal1*-reporter expression is regulated in a circadian dependent manner (Figure 5A). Using the *Bmal1*-reporter system, we found that treatment with 10 μM SR8278, a small-molecule REV-ERB antagonist previously shown to increase circadian rhythms in nonskin tissues (24), enhanced the amplitude of rhythmic BMAL1 activity (Figure 5, A and B).

To test if circadian augmentation has an antiviral effect, we used surgically discarded human skin samples. We separated human epidermis from dermis, infected the epidermis ex vivo with HSV-1 in an air–liquid interface culture system, and treated the epidermal explant culture with either vehicle control or 10 μM SR8278 for 24 hours. By immunofluorescent staining and quantification, we observed that SR8278 treatment significantly reduced HSV-1 antigen expression in the epidermis (Figure 5, C and D). We verified this reduction via qPCR for HSV-1 UL29 gene with human K14 gene used for internal control (Figure 5E).

We then asked whether the antiviral effect of SR8278 was dependent on circadian and antiviral factors. We performed siRNA-mediated gene silencing of BMAL1 in *NTERT* keratinocytes and visualized HSV-1 using immunofluorescence after treatment with SR8278 (Figure 5F). After controlling for cell number by nuclei staining using Fiji (ImageJ), we found that SR8278’s antiviral effect was lessened in si*BMAL1* transfected cells (Figure 5G). To verify these findings, we infected *NTERT* keratinocytes with siRNA-mediated gene silencing of *BMAL1* and *CLOCK*. qPCR analysis of the viral gene UL29 revealed that *BMAL1* and *CLOCK* gene silencing significantly increased virus in culture media of SR8278-treated cells (Figure 5H), indicating that SR8278’s antiviral effect is predicated on circadian function. Interestingly, when we suppressed AVPs OAS1 and IFITM1 via siRNA in *NTERT* keratinocytes, SR8278’s effect was also lessened (Supplemental Figure 7), suggesting SR8278’s antiviral effect is both circadian and AVP dependent.

We subsequently examined if other circadian-augmenting compounds would have antiviral effects. Nobiletin is an antioxidant flavonoid with multiple pharmacological effects, including antioxidant properties (25), as well as an ROR agonist that potentiates circadian rhythms (14). Using the *NTERT Bmal1*-luciferase cell line, we verified that nobiletin increased *Bmal1*-reporter expression in keratinocytes (Supplemental Figure 8A). Treatment with nobiletin decreased HSV-1 infection of human epidermal explants (Supplemental Figure 8, B–D), suggesting an antiviral activity of circadian enhancers.

### Viral infections in aging skin are reduced by circadian augmentation.

**Figure 6:**
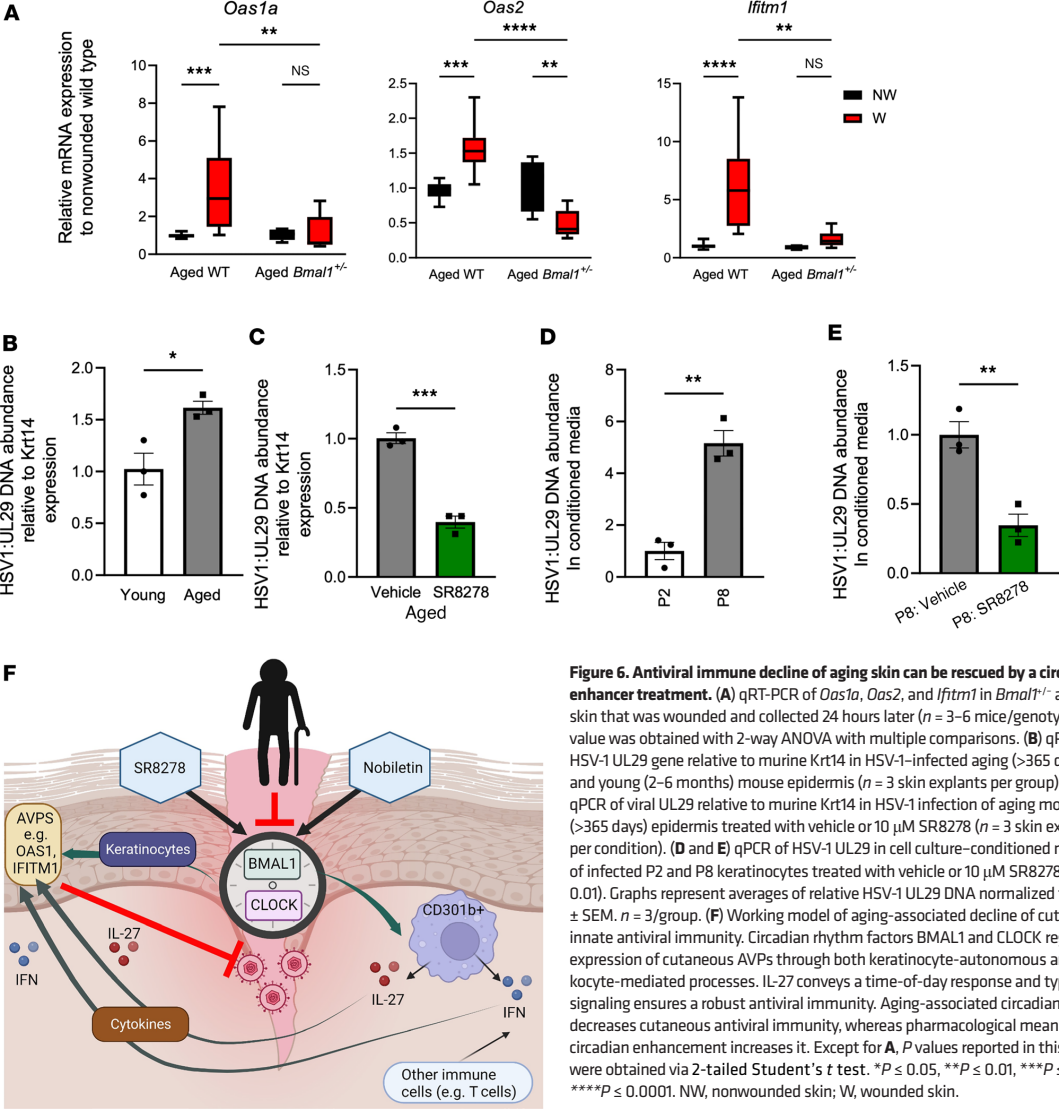
Antiviral immune decline of aging skin can be rescued by a circadian enhancer treatment. (**A**) qRT-PCR of *Oas1a*, *Oas2*, and *Ifitm1* in *Bmal1*^+/–^ and WT skin that was wounded and collected 24 hours later (*n* = 3–6 mice/genotype). *P* value was obtained with 2-way ANOVA with multiple comparisons. (**B**) qPCR of HSV-1 UL29 gene relative to murine Krt14 in HSV-1–infected aging (>365 days) and young (2–6 months) mouse epidermis (*n* = 3 skin explants per group). (**C**) qPCR of viral UL29 relative to murine Krt14 in HSV-1 infection of aging mouse (>365 days) epidermis treated with vehicle or 10 μM SR8278 (*n* = 3 skin explants per condition). (**D** and **E**) qPCR of HSV-1 UL29 in cell culture–conditioned medium of infected P2 and P8 keratinocytes treated with vehicle or 10 μM SR8278 (MOI, 0.01). Graphs represent averages of relative HSV-1 UL29 DNA normalized to Krt14 ± SEM. *n* = 3/group. (**F**) Working model of aging-associated decline of cutaneous innate antiviral immunity. Circadian rhythm factors BMAL1 and CLOCK regulate expression of cutaneous AVPs through both keratinocyte-autonomous and leukocyte-mediated processes. IL-27 conveys a time-of-day response and type I IFN signaling ensures a robust antiviral immunity. Aging-associated circadian decline decreases cutaneous antiviral immunity, whereas pharmacological means of circadian enhancement increases it. Except for **A**, *P* values reported in this figure were obtained via 2-tailed Student’s *t* test. *P ≤ 0.05, **P ≤ 0.01, ***P ≤ 0.001, ****P ≤ 0.0001. NW, nonwounded skin; W, wounded skin.

Aging skin is subject to immunosenescence and is susceptible to viral infections. Thus, we examined if circadian modulation has antiviral activity in aging skin. We found that 1-year-old, *Bmal1*^+/–^ mutant mouse aging skin, which displays premature aging, also shows deficiency of AVP transcription in the wound environment (Figure 6A). Following this pattern of antiviral deficiency, we infected epidermal explants of *Bmal1*^+/–^ and WT animals with HSV-1. By qPCR, we found that HSV-1 levels were higher in *Bmal1*^+/–^ epidermis than in WT counterparts (Supplemental Figure 9). Next, we examined the effects of aging on viral infection. We infected aged and young WT murine epidermal explants with HSV-1 and found via qPCR that HSV-1 levels were higher in aged skin than in younger skin (Figure 6B). Treatment with the circadian drug SR8278 reduced HSV-1 viral load in aged skin by approximately 50% as measured by qPCR (Figure 6C). Finally, we tested infection susceptibility of passaged human keratinocytes as a pseudoaging model. We found that human primary keratinocytes at P8 produced more virus than did P2 keratinocytes (Figure 6D) and that this increased viral replication could be suppressed by treatment of SR8278 (Figure 6E). These data indicate that circadian-augmenting agents have antiviral effects on aging skin (Figure 6F).

## Discussion

Our data reveal a pharmacologically tractable model of age-mediated circadian regulation of antiviral immunity of the skin. Skin aging leads to decline of circadian function in the skin, compromising epidermal and dermal antiviral responses. Pharmacological agents that potentiate skin-cell circadian amplitude improve immunity via AVP effect. Our data delineate new mechanisms responsible for the immunosenescent decline of antiviral immunity in aging skin, underscoring the circadian pathway as a new therapeutic target for enhancing aging skin barrier function.

Previous studies have shown that IFN-stimulated genes are affected by skin circadian rhythms through TLR7-dependent stimulation (12). We have demonstrated that type I IFN signaling is required for activation of antiviral immune responses but may not convey time-of-day responses. However, type I IFN signaling is required for maximum AVP expression irrespective of time of day, suggesting that the IFN pathway as the primary regulator of AVPs is subject to regulation by circadian factors. In this regard, STAT1 and STAT3, transcription factors involved in IFN response, display time-of day-responses (26) and could be an indirect regulatory step between circadian transcription factors and AVPs. Circadian factors may directly regulate AVPs by binding to the E-box consensus elements, which are present in gene promoters of antiviral genes such as *Oas1*, *Oas2*, and *Ifitm1*. Such possibilities may be explored via skin cell–specific ChIP-Seq for BMAL1 and CLOCK (27). These experiments may also help explain why certain AVPs in primate skin follow distinct temporal expression, because AVPs may experience differential BMAL1/CLOCK binding efficacies in the skin.

Our data show that CD301b^+^ leukocyte-derived IL-27 is important for the time-of-day–dependent response of AVP expression. It will be important to determine whether the decreased dermal infiltration of CD301b^+^ leukocytes in aged skin and *Bmal1^–/–^* skin is a result of central or local circadian decline and if rescuing this defect can restore circadian AVP functionality. Knowing specifically how the circadian rhythm acts on circulating immune cells and skin resident cells will provide better insights into how to leverage circadian rhythms to improve cutaneous tissue regeneration and defense against infection in aging skin. This information may also prove useful in understanding skin infections that resist traditional IFN-induced immunity, such as monkey pox (28).

We focused on a common skin pathogen, HSV-1, but intriguingly, different viruses may interplay with circadian rhythms in distinct fashions. For example, respiratory syncytial and vesicular stomatitis virus replication rates had opposite responses to circadian deletion (7), possibly due to how these viruses differ in viral entry and replication machinery. Other work has shown that time of day affects response to HSV-2 in murine skin, as well as treatment responses (29). Our data show that siRNA-mediated gene silencing of *BMAL1*/*CLOCK* sensitized human keratinocytes to HSV-1 infection and diminished protective effects conferred by pharmacological circadian enhancers (SR8728 and nobiletin). In agreement with our data, *Bmal1^–/–^* mice exhibit greater HSV-1 viral replication than do their WT counterparts (6), which is attributed, in part, to aspects of host cell–virus interaction, such as intracellular trafficking and chromatin assembly. Paradoxically, BMAL1 and CLOCK are found to be hijacked by viral proteins to support viral replication (30). How to best balance the antiviral and pro-viral activity of circadian factors requires further study and should incorporate more skin-trophic viruses that have pandemic-level infectivity risks (31).

In summary, we demonstrate a mechanism of aging-associated skin infection risk. Age leads to circadian suppression in the skin, ultimately triggering a reduced antiviral barrier function in a BMAL1/CLOCK-dependent manner. Circadian pharmacological agents can rescue age-related viral susceptibility in the skin, suggesting a therapeutic pathway for combatting immunosenescence. Our findings have potentially wide implications for aging skin and may lead to new treatment strategies for prevention of cutaneous infection, wound care, and overall skin health in aging populations.

## Methods

### In vivo wounding experiments.

C57BL/6 WT, *Ifnar1^–/–^*, B6.129-Arntltm1Bra/J (*Bmal1^–/–^*), ClockΔ19 mice (21), and LysM-Cre mice (21, 32) were obtained from Jackson Laboratory. *IL27p28^fl/fl^* mice were gifts from Zhinan Yin (Biomedical Translational Institute, Jinan University, Guangzhou, Guangdong Province, China) and Li Fan Lu (UCSD, San Diego, California, USA). All mice were maintained under a specific pathogen–free environment. After anesthesia of the mice, 3 mm punch wounds were made on the back of each mouse at distinct times of day and collected 24 hours after wounding. Tissue from nonwounded and wounded skin was dissected from each mouse; lysed in TRIzol reagent (Thermo Fisher Scientific); and kept at –80°C for RNA extraction, placed in OCT for immunofluorescence, or immunostained for flow cytometry. Information regarding RNA extraction, qRT-PCR, immunofluorescence, or flow cytometry approaches can be found in Supplemental Methods. For aged and young skin studies, wounds were inflicted between 8 am and 9 pm. Young mice were aged between 3 and 6 months, and elderly mice were older than a year. Male and female mice were used in this study.

### Keratinocyte cell culture.

Human primary keratinocytes were purchased from Thermo Fisher Scientific. Cells were grown in a 37°C incubator in serum-free Epi-Life cell culture medium (Gibco) supplemented with Epi-Life Defined Growth Supplement containing 0.05 mM Ca^2+^. *NTERT 2G* keratinocytes were a gift from the laboratory of Johann Gudjonsson (University of Michigan, Ann Arbor, Michigan, USA) and cultured in Keratinocyte SFM medium supplemented with EGF and BPE (Gibco) prior to use for luciferase and infection studies. Complete keratinocyte-associated methods, including circadian synchronization, siRNA experiments, and infections, can be found in Supplemental Methods.

### RNA-Seq gene expression and microarray gene expression data.

Publicly available, nonhuman primate, RNA-Seq gene expression data were downloaded from the Gene Expression Omnibus (GSE98965). The data were provided as a normalized expression matrix as calculated by Mure et al. (20). Publicly available microarray gene expression data from Geyfman et al. (19) were downloaded from the Gene Expression Omnibus (33) (GSE38625). Further computational analysis methods can be found in Supplemental Methods.

### HSV staining and qPCR.

For a full description of viral infection, please see Supplemental Methods. Briefly, human or murine epidermis were maintained in keratinocyte growth medium and infected with 10,000 focus-forming units per sample of HSV-1 strain NS in the presence of vehicle, nobiletin, or SR8728 (Sigma) at 5–10 μM. The epidermis was then either placed in 4% formaldehyde to fix overnight and subsequently stained for HSV-1 antigen or was lysed for DNA extraction and viral quantification.

### Statistics.

All statistical tests were performed in GraphPad Prism. Throughout figures, data in box-and-whisker plots are shown as 10th to 90th percentile. Data in scatterplots with scale bars are shown as mean ± SEM. *P* values ≤ 0.05 were considered statistically significant.

### Study approval.

Animal procedures were performed in agreement with the recommendations in the *Guide for the Care and Use of Laboratory Animals* of the NIH (National Academies Press, 2011). Animal protocols were approved by Duke University’s IACUC (Animal Welfare Assurance). Human tissue was used in accordance with Duke IRB approval.

### Data availability.

All data needed to evaluate our conclusions are present in the paper and/or the supplement. Furthermore, all data points shown in graphs and values behind any reported means are provided in our Supporting Data Values file. All data and materials generated in this study will be made available upon request and completion of the material transfer agreement per requirement by the original provider of cell lines and animal models. Murine skin (GSE38625) and baboon skin (GSE98965) data sets are publicly available (19, 20).

## Author contributions

SJK, VL, ASM, DH, and JYZ conceptualized the study; SJK, VL, JLS, ASM, JYZ, JC, DE, and DC contributed to methodology; SJK, VL, PTK, MP, DC, ASM, JYZ, and DKW contributed to the investigation; SJK and VL conducted the visualization; JYZ, ASM, and JC acquired funding for the study; JYZ, ASM, JC, and KD contributed to project administration; JYZ, ASM, JC, and KD supervised the study. All authors contributed to the writing, reviewing, and editing of the manuscript.

## Supplementary Material

Supplemental data

Supporting data values

## Version 1 09/19/2023

In-Press Preview

## Version 2 10/23/2023

Electronic publication
